# Interface dynamics explain assembly dependency of influenza neuraminidase catalytic activity

**DOI:** 10.1080/07391102.2013.855142

**Published:** 2013-11-27

**Authors:** Susanne von Grafenstein, Hannes G. Wallnoefer, Johannes Kirchmair, Julian E. Fuchs, Roland G. Huber, Michaela Schmidtke, Andreas Sauerbrei, Judith M. Rollinger, Klaus R. Liedl

**Affiliations:** ^a^ Institute of General, Inorganic and Theoretical Chemistry and Center for Molecular Biosciences Innsbruck (CMBI), University of Innsbruck, Innsbruck, Austria; ^b^ Department of Chemistry, Unilever Centre for Molecular Sciences Informatics, University of Cambridge, Cambridge, UK; ^c^ Department of Virology and Antiviral Therapy, Jena University Hospital, Jena, Germany; ^d^ Institute of Pharmacy, Pharmacognosy and CMBI, University of Innsbruck, Innsbruck, Austria

**Keywords:** influenza, neuraminidase, molecular dynamics simulation, protein assembly, oligomerization

## Abstract

Influenza virus neuraminidase (iNA) is a homotetrameric surface protein of the influenza virus and an established target for antiviral drugs. In contrast to neuraminidases (NAs) of other biological systems (non-iNAs), enzymatic activity of iNA is only observed in a quaternary assembly and iNA needs the tetramerization to mediate enzymatic activity. Obviously, differences on a molecular level between iNA and non-iNAs are responsible for this intriguing observation. Comparison between protein structures and multiple sequence alignment allow the identification of differences in amino acid composition in crucial regions of the enzyme, such as next to the conserved D151 and the 150-loop. These differences in amino acid sequence and protein tetramerization are likely to alter the dynamics of the system. Therefore, we performed molecular dynamics simulations to investigate differences in the molecular flexibility of monomers, dimers, and tetramers of iNAs of subtype N1 (avian 2004, pandemic 1918 and pandemic 2009 iNA) and as comparison the non-iNA monomer from *Clostridium perfringens*. We show that conformational transitions of iNA are crucially influenced by its assembly state. The protein–protein interface induces a complex hydrogen-bonding network between the 110-helix and the 150-loop, which consequently stabilizes the structural arrangement of the binding site. Therefore, we claim that these altered dynamics are responsible for the dependence of iNA’s catalytic activity on the tetrameric assembly. Only the tetramerization-induced balance between stabilization and altered local flexibility in the binding site provides the appropriate arrangement of key residues for iNA’s catalytic activity.

## Introduction

The enzyme class of neuraminidases (NAs), EC 3.2.1.18, unifies exo-sialidases cleaving the glycosidic bonds of terminal sialic acids from carbohydrates, glycolipids, or glycoproteins. One of the most thoroughly studied NAs is the influenza virus NA (iNA) (Air, 2012; Gamblin & Skehel, 2010; Grienke et al., 2012). In the viral life cycle, iNA is responsible for cleaving mature virus particles from the host cell. This role is complementary to the function of the second antigenic surface structure, hemagglutinin, which binds to the sialic acid receptor on the host cell to trigger virus entry. iNA destroys the hemagglutinin receptor and reduces the binding sites for the pathogen on the surface of a host cell. Thereby, it facilitates the detachment of the mature virus from infected cells and prevents virus aggregation. Inhibition of iNA with zanamivir or oseltamivir limits infection rates, as the enzyme is essential for the spread of the virus.

NAs are also present in other biological systems, such as bacteria, fungi, protozoa, and mammalia. These non-influenza neuraminidases (non-iNAs) are critical factors for virulence or play a role in metabolism and cell differentiation (see references Schwerdtfeger and Melzig (2010) and Kim, Oh, Kang, and Kwon (2011) for reviews on non-iNAs). For example, the pathogen *Clostridium perfringens* has several sialidases, which are essential for the nutrition of the bacterium (Newstead et al., 2008).

Within the glycoside hydrolases (GH) classification, NAs form one clan characterized by a common six-blade β-propeller fold around their active site (Davies & Henrissat, 199; Henrissat & Bairoch, 1996). The clan comprises GH family 33 (non-iNAs) and GH family 34 (iNAs), which differ in their protein sequences. Furthermore, several residues directly involved in the catalytic reaction have similar positions in members of both families, as determined by X-ray crystallography (Taylor, 1996). The common structure of iNAs and non-iNAs is conserved up to the tertiary level. However, their quaternary structures are distinct. iNAs are homotetramers by assembly of the catalytic domain, while most non-iNAs are monomers or associate to oligomers via adjacent protein domains. For example, the non-iNA trans-sialidase in *Trypanosoma* species is an oligomer of which the isolated monomeric catalytic domain is still active (Schenkman, Chaves, Decarvalho, & Eichinger, 1994). In contrast to that, iNA needs the tetramerization to be catalytically active (Air, 2012).

Nine subtypes of iNA cluster in two groups by their sequence identity: group 1 comprises the subtypes N1, N4, N5, N8, and group 2 comprises of N2, N3, N6, N7, N9 (Russell et al., 2006). The tetrameric character of iNA was first suggested for subtype N2 and was identified as the biologically active unit in 1972 (Bucher & Kilbourne, 1972). The iNA homotetramer forms spikes of a mushroom-like shape anchored to the membrane with one helix for each subunit (Air, 2012; Air & Laver, 1989). The structure of catalytic head domain of iNA has been elucidated by X-ray crystallography (Air, 2012; Air & Laver, 1989). In the iNA head, the secondary and quaternary structures of the four subunits situated around a C4 symmetry axis are conserved for all subtypes (Varghese, Laver, & Colman, 1983). In contrast to the classical iNAs, the NA-like N10 protein of a recently discovered H17N10 influenza A virus, isolated in bats, was shown to crystallize in a monomeric and a tetrameric form. Besides this monomer no structural insights into iNA monomers are available (Li et al., 2012).

In crystal structures of virus subtypes N2 and N9, a glycosylation motive at N200 interacts with the neighboring subunit and is supposed to contribute to the stability of group 2 iNA tetramers (Air, 2012). However, this glycosylation site is not conserved in group 1 iNAs (Xu, Zhu, Dwek, Stevens, & Wilson, 2008). A single point mutation of the active site glutamate E119 into glycine was observed to induce disintegration of the tetramer assembly in N9 (Colacino et al., 1997). Loss of a salt bridge between E119 and the conserved R156 is supposed to mediate the link between active site and tetramer interface (Colacino et al., 1997). For subtype N1 iNA a systematic investigation of stalk length variations identified both transmembrane region and the catalytic head as factors contributing to the tetramer assembly (da Silva, Nordholm, Madjo, Pfeiffer, & Daniels, 2013). An analysis of the 1918 pandemic N1 confirmed that iNA indeed requires tetramer assembly to exhibit enzymatic activity (Wu, Ethen, Hickey, & Jiang, 2009). The importance of tetramerization is further emphasized by the efforts to develop a plasmid expression platform for recombinant iNA with a suitable tetramerization domain in order to stabilize the quaternary structure (Schmidt, Attwood, Mohr, Barrett, & McKimm-Breschkin, 2011). However, an explanation for iNA tetramerization is still missing and the mechanism of how it affects catalytic activity remains unclear (Air, 2012).

Homo-assembly of proteins is frequently observed and has a wide range of biological implications (Hashimoto & Panchenko, 2010; Levy, Erba, Robinson, & Teichmann, 2008). Protein oligomerization is assumed to stabilize the structural and thermodynamic integrity of the individual subunits and also enables cooperative communication between the subunits and mediation of allosteric effects (Ali & Imperiali, 2005; Goodsell & Olson, 2000). Amaro et al. (2007) investigated different possible consequences of oligomerization of iNA applying molecular dynamics (MD) simulations. Their MD simulations of the tetrameric N1 iNA indicated that the dynamics of the four subunits are independent from one another. By comparing simulations of a monomer and a tetramer, Amaro et al. (2007) identified a secondary structure element sensitive to the assembly state. The α-helix around S105–S110 was shown to be unstable when iNA was simulated as a monomer. The 110-helix forms a part of the protein–protein interface and is located in a distal region of the protein not linked to the active center. These observations suggest a stabilizing effect of the protein fold by inter-subunit contacts present in the fully oligomerized state. However, the connection between the assembly state and enzymatic activity has not been elucidated so far.

As NA homologs originating from different biological origins have substantial differences with respect to their biologically active unit, they are predisposed for studying the reasons and specifics of quaternary assembly. In this work, we aim to find an explanation for the sensitivity of iNAs to their assembly state. We performed a structure-guided sequence alignment with two iNAs and six non-iNAs. The non-iNAs cover three NAs of bacterial organisms and three of eukaryotic organisms. Subsequently, we employed MD simulation techniques to evaluate the influence of oligomerization on the protein dynamics, with special focus on an iNA-specific structural feature identified by sequence alignment. In contrast to experimental studies, this theoretical approach allows a direct transfer of the investigated systems into different assembly states. We investigated monomer, dimer, and tetramer state of three apo structures of iNA subtype N1, pandemic N1 from 1918 (1918iNA), avian N1 from 2004 (2004iNA) and pandemic N1 from 2009 (2009iNA). The latter iNA was simulated in zanamivir-bound state (2009iNA + ligand) to investigate ligand binding. As representative for monomer non-iNAs the enzyme of the bacterium *C. perfringens* was selected (non-iNA Cp) in apo state and ligand-bound (non-iNA Cp + ligand).

## Materials and methods

### Sequence and structure comparison of NA homologs

Ten X-ray structures of NAs of different biological systems (Table 1) are studied here by a structure-guided sequence analysis. Four representative iNAs, covering the subtypes N1 and N2, which are commonly isolated in humans, were compared to six non-iNAs. Superpositions was performed in MOE, version 2011.10, (Chemical Computing Group, 2011) using the Protein Align module. Thereby, constraints were applied on a set of six-conserved binding site residues: R118, R292, R371, Y406, E277, and D151 (amino acid numbering according to iNA). Pair-wise sequence identities as calculated in MOE are summarized in Table S1. The resulting alignments were visualized using Jalview, version 2.6.1 (Waterhouse, Procter, Martin, Clamp, & Barton, 2009). NA structures are visualized with PyMOL, version 1.3 (Schrödinger, 2010).

**Table 1.** NAs of different biological origins used in this study.

Code	PDB^a^	Source organism
1918iNA N1	3beq	Influenza A virus, H1N1
		A/Brevig Mission/1/1918
2004iNA N1	2hty	Influenza A virus, H5N1
		A/Vietnam/1203/2004
2009iNA N1	3nss/3ti5	Influenza A virus, H1N1
		A/California/04/2009
1967iNA N2	1ivc	Influenza A virus, H2N2
		A/Tokyo/3/1967
Non-iNA Cp	2vk6	*C. perfringens*
		(bacteria)
Non-iNA Sp	2vw1	*Streptococcus pneumoniae*
		(bacteria)
Non-iNA Mv	1eus	*Micromonospora vidifaciens*
		(bacteria)
Non-iNA Tc	1ms0	*Trypanosoma cruzi*
		(protozoa)
Non-iNA Af	2xzi	*Aspergillus fumatus*
		(fungi)
Non-iNA Hs	1vcu	*Homo sapiens*
		(mammalia)

^a^ Structural information is accessible at www.pdb.org (Berman et al., 2000).

### Preparation of structures for MD simulations

Three iNA systems in apo state and one ligand-bound iNA of subtype N1 were investigated by MD simulations using X-ray structures as starting coordinates. Apo X-ray structures were selected for 1918iNA (PDB 3bqe), 2004iNA (PDB 2hty), and 2009iNA (PDB 3nss). For these iNA variants monomer, dimer, and tetramer systems were prepared using chain arrangements taken from crystals as summarized in Table S2 (Supporting Information). The ligand-bound state of iNAs was studied by the example of 2009iNA with zanamivir co-crystallized in the monomer and tetramer states (PDB 3ti5). As representative for monomer non-iNAs the enzyme of the bacterium *C. perfringens* was selected (non-iNA Cp). A ligand-free simulation as well as a ligand-bound simulation were prepared from the X-ray structure co-crystallized with the inhibitor 2-deoxy-2,3-dehydro-N-acetyl neuraminic acid abbreviated as DANA (PDB 2vk6). The construction of a non-iNA in an artificial tetrameric state, as suggested by a reviewer, is not possible as the monomers do not provide the corresponding interfaces.

All water molecules present in the crystal structure were retained. In line with previous studies of iNA (Amaro, Cheng, Ivanov, Xu, & McCammon, 2009; Amaro et al., 2007; Grienke et al., 2010), co-crystallized organic molecules other than ligands in the active site were removed. The structurally important Ca^2+^ ion bound to residues 379–390 was imported from the structure of 1918iNA to 2004iNA as it is missing in this X-ray structure (Xu et al., 2008). The Ca^2+^ ion and the Mg^2+^ ions co-crystallized in the non-iNA Cp were retained for the simulation.

Protonation at pH = 7 was performed with the Protonate3D tool (Labute, 2009), as implemented in MOE 2010.10 (Chemical Computing Group, 2011). For all iNAs and all their assembly states H144 was protonated as δ-H-isomer. Finally, all systems were soaked in octahedral boxes of TIP3P water molecules applying the LEAP tool of Amber10 (Case et al., 2008) with a minimum distance of 10 Å between protein and box wall.

### MD simulation setup and protocol

Force field parameters were applied from the Amber force field ff99SB for the protein residues and Mg^2+^ ions (Hornak et al., 2006), and from Bradbrook et al. (1998) for Ca^2+^ ions. Parameters for the ligands zanamivir and DANA were derived from GAFF (Wang, Wolf, Caldwell, Kollman, & Case, 2004) and point charges were calculated by RESP fitting (Bayly, Cieplap, Cornell, & Kollman, 1993) of the electron density derived at HF-6/31G*-level using Gaussian09 (Frisch et al., 2009).

A cut-off value of 8 Å was set for non-bonded interactions. Particle Mesh Ewald (PME) (Darden, York, & Pedersen, 1993) was used for long-range electrostatics (tolerance 0.00001, Ewald coefficient 0.34864 Å^−1^). Bonds to hydrogen atoms were fixed with the SHAKE algorithm (Miyamoto & Kollman, 1992). Temperature was controlled using the Langevin algorithm (Wu & Brooks, 2003) with a collision frequency of 2.0 ps^−1^.

The proteins were equilibrated as described earlier (Wallnoefer, Liedl, & Fox, 2011; Wallnoefer, Lingott, Gutierrez, Merfort, & Liedl, 2010), applying the Sander module of Amber10 (Case et al., 2008). Hydrogen positions were optimized (500 steps steepest descent, 500 steps conjugate gradient) with position restraints on heavy atoms (1000 kcal/mol Å^2^). Subsequently, water positions were optimized with position restraints on protein heavy atoms (1000 kcal/mol Å^2^). Afterwards, 100 ps of gradual heating (NVT), 200 ps NPT simulation for box size adaption, and 100 ps of gradual annealing were performed with positional restraints (1000 kcal/mol Å^2^) on protein heavy atoms to disorder the solvent box. The energy of the protein was gradually minimized with decreasing positional restraints (from 1000 to 0 kcal/mol Å^2^) on protein heavy atoms in 13 stages of 500 steps steepest descent and 500 steps conjugate gradient each. Finally, the system was heated from 100 to 300 K over 400 ps (NVT). Constant pressure and temperature (300 K) were applied for the production runs using the module PMEMD. In case of the 2009iNA monomer, four productive simulations were run from four different seeds for random number generator in order to obtain sampling which is comparable to the tetramer. The first two ns of each simulation completed the equilibration. Frames were stored every 0.5 ps to trajectories of 28 ns for analysis.

### Analysis of the MD trajectories

The trajectories were analyzed using PTRAJ, which is part of from AmberTools 1.4 (Case et al., 2008). Root-mean square deviations (RMSDs) of all registered snapshots to crystal structures were calculated for backbone heavy atoms. The RMSD analysis was performed on the complete assemblies as well as on a subunit-wise level to ensure comparable sampling. RMSD calculations were also performed for every 400th frame of the monomer and tetramer simulations to compare the sampled conformations with one another, resulting in a two-dimensional heat map (2D-RMSD plot). N-terminal and C-terminal residues were neglected to avoid effects from insufficiently sampled motions and the additional residues resolved for 2009iNA; hence, the 2D-RMSD heat map was generated on residues 85–461 consistently for all iNA systems. For 2D-comparison of active site geometries, the definition of 61 cavity residues was adopted from earlier work (residues 114–119, 134–140, 145–152, 156, 178–180, 222–227, 244–246, 276–277, 292, 294, 347–350, 371, 403–406, 423, 425–432, 437–441) (Grienke et al., 2010). Principal component analysis (PCA) was performed for the covariance metrics of C-positions of snapshots of individual subunits extracted for every 400th frame of the trajectories to compare the sampled conformational space. The first two principal components (PCs) representing the ones with the two highest eigenvalues were used for projection of the snapshots. Positional fluctuations of backbone atoms were calculated residue-wise as B-factors over all frames. Hydrogen bonds were registered for each ps if the distance between donor and acceptor was <3 Å and the angle between hydrogen acceptor, hydrogen, and hydrogen donor was >120°. For comparability reasons, all hydrogen bonds formed by topologically equivalent side chain atoms were summed up. For inter-subunit hydrogen bonds, average occupancy rates were calculated for the four equivalent interfaces of the tetramers. All inter-subunit hydrogen bonds occurring with an occupancy rate of at least 5% in one of the simulated systems were further investigated.

## Results

### Sequence alignment of NAs of different origin: identification of iNA-specific insertions

**Figure 1.**
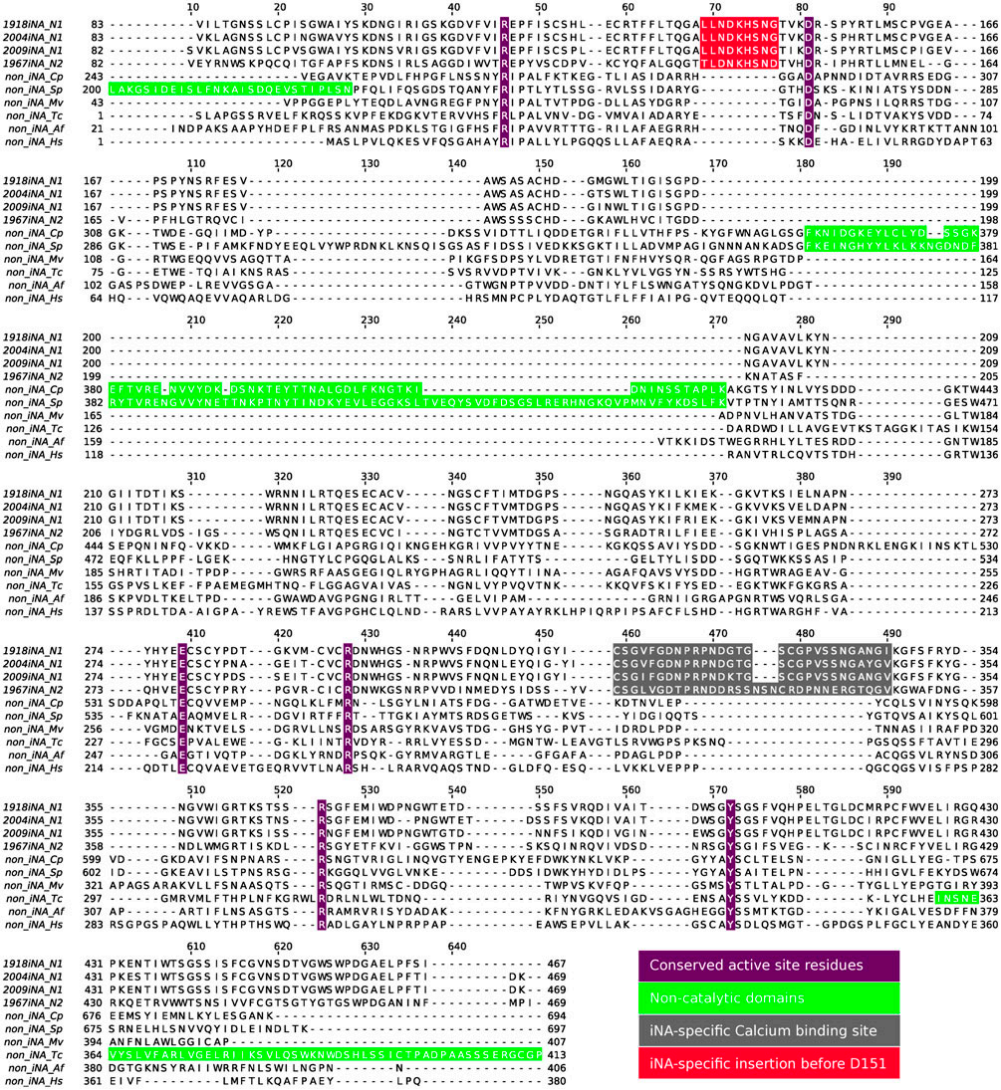
Structure-guided sequence alignment of four iNAs and six non-iNAs based on conserved active site residues (violet) reveals two iNA-specific loop insertions: nine amino acids residues 138–147 (red) preceding the conserved aspartic acid D151 and the Ca^2+^ ion binding site between residues 320–350 (grey). The focus is on the catalytic domains of NAs, therefore parts of non-catalytic domains (green) are not shown (residues 1–199 in non-iNA Sp and residues 414–634 in non-iNA Tc).

Structure-guided sequence alignment of four iNAs and six non-iNAs of different biological origins (Table 1) was constrained toward a structural superposition of active site residues known to be conserved among all investigated NAs (Figure 1). Thus, structurally equivalent regions can be identified despite the low sequence identity between iNA and the non-iNAs. Sequence identities among non-iNAs range from 8 to 27% for the catalytic domains, whereas sequence identity between iNAs and non-iNAs is below 10% (Table S1). The comparison of the two iNA subtypes N1 vs. N2 show a sequence identity of around 36%. The subtype N1 representatives differ by 10% of the amino acids. We find the non-iNAs to be more closely related to one another than to iNAs, which is in agreement with systematic sequence studies of GH families (Davies & Henrissat, 1995).

**Figure 2.**
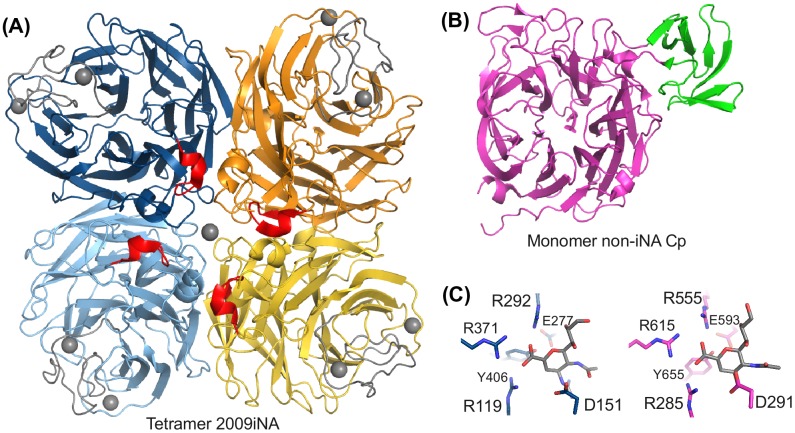
NA folds of (A) Tetrameric iNA with iNA-specific insertions highlighted in red (insertion before D151) and gray (Ca^2+^ ion binding site). (B) Bacterial NA of *C. perfringens* (non-iNA Cp) with the non-catalytical domain in green. (C) Respective active sites with ligands (gray) zanamivir in iNA (blue) and non-iNA Cp (pink). Six conserved active site residues are shown in stick representation. Structures of NAs from further investigated systems are shown in Figure S1.

Three-dimensional superposition of the protein structures shows six-conserved binding site residues common to the active sites of all NAs (marked in violet in Figure 1): three-conserved arginines, R119, R292, R371 (amino acid numbering according to iNA), form the pocket accommodating the carboxylic acid moiety of the sialic substrate and iNA inhibitors (Figures 1 and 2). Also, Y406 and E277 are conserved (Figure 1). These active site residues, which are involved in the catalytic mechanism in non-iNAs (Newstead et al., 2008; Telford et al., 2011), are packed within the central part of the β-propeller, located in conserved secondary structure elements. The last of the six-conserved active site residues, D151, is found in the loop region between the second and the third β-strand of the first β-sheet within the propeller architecture (Figures 2 and S1 showing the structures of NAs of other biological systems).

The aspartic acid D151 and equivalents in non-iNAs are conserved in the amino acid sequence and they also superpose at the structural level. Nevertheless, a remarkable difference in the sequence context of D151 in iNAs compared to non-iNAs can be identified: in the case of iNAs, the loop between the two β-strands consists of 20 amino acid residues, while the equivalent loop of non-iNAs is only half as long (Figure 2(A) and (B)). The loop insertion in iNAs has a short helical element and is in close spatial proximity to the neighboring subunit in the tetrameric arrangement (Figure 2(A)). There is no analog of this elongation (comprising residues 138–147) present in non-iNA sequences (sequence and structural fragments marked in red in Figures 1 and 2); hence, this loop extension appears to be a typical feature of iNAs.

A characteristic distinguishing iNAs from non-iNAs is an additional loop extension, which links the fourth and the fifth propeller sheet. Including the insertion this loop extends in total over 30 residues in iNAs (Figure 1, residue 320 and 350 highlighted in gray). Structurally, this region forms a conserved binding site for a Ca^2+^ ion located next to the active site (Figures 2(A) and S1).

In contrast, in the outermost β-strand of the second sheet, in the proximity of residue 200 (iNA numbering), non-iNAs show various insertions compared to iNAs. For example, the bacterial non-iNAs Cp and Sp have non-catalytic domains, such as lectin binding domains, inserted (Figures 1 and 2(B) and S1 highlighted in green) and in human non-iNA two small insertions form short helices at this sequence position.

### Structural comparison of simulations: conformational flexibility of iNAs depends on the assembly state

The monomer, dimer, and tetramer structure of three N1 subtypes (2004iNA, 1918iNA, 2009iNA) was simulated in their ligand-free state. In addition, 2009iNA was simulated in presence of the inhibitor zanamivir (2009iNA + ligand) in order to investigate the effect of ligand binding on protein dynamics. Non-iNAs were investigated by the example of *C. perfringens* NA, which was simulated in ligand-free (non-iNA Cp) and ligand-bound state (non-iNA Cp + ligand).

Analysis of the (heavy atom) backbone RMSD indicates that all simulated systems were stable over the full simulation time and that structural integrity of the assembly is warranted (Figure S2). Analysis of the individual subunits allows the comparison of systems with equivalent sampling showing convergence with RMSD values below 2.2 Å (Figure S2). Despite these low deviations from the initial structures, a clear influence of the structural assembly on dynamic behavior of the individual subunits can be observed for iNAs: RMSD values decrease with higher assembly states, meaning that subunits in dimer simulations show lower mean RMSDs compared to monomer simulations. Also, subunits of tetramers show smaller structural deviations compared to dimers (see Table S3, which summarizes the RMSD means and standard deviations for all simulations).

**Figure 3.**
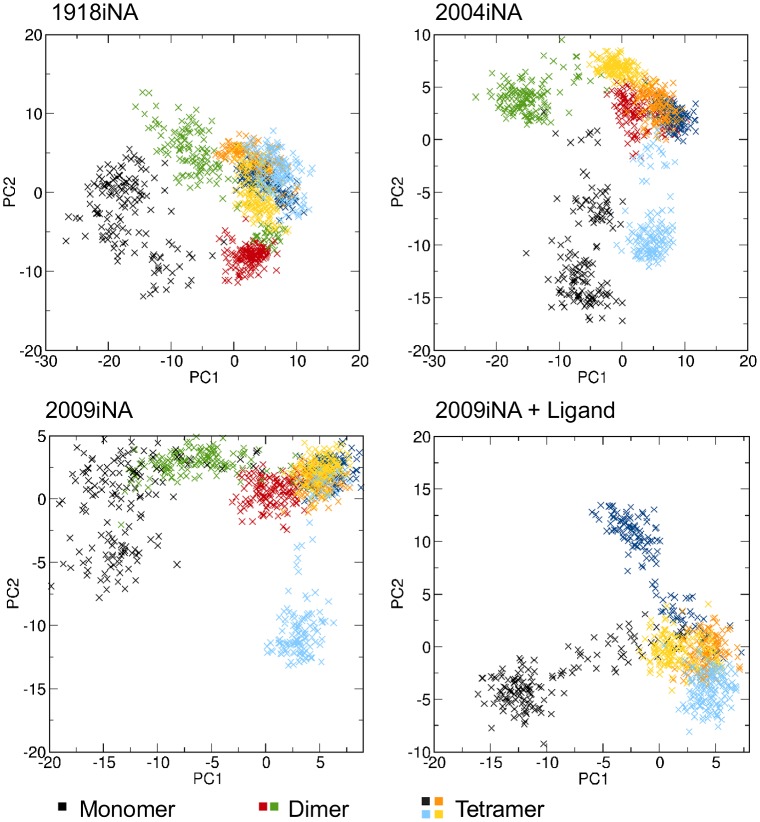
PCA shows that PC1 reflects the difference between the assembly states. Snapshots from monomer simulations (black) and subunit 2 in the dimer simulation (green) are separated along the axis of PC1 in the projection. PC2 reflects loop movements within the individual subunits.

To investigate the differences between the assembly states, a PCA of the C-α position was performed based on the subunits of all investigated assembly states. The first two PCs (PC1 and PC2) were used for projection of the snapshots (Figure 3). The four subunits from the tetramer cluster, whereas the monomer and dimer snapshots sample a distinct area defined by the first PCs. Consistently, for all iNA systems PC1 separates the assembly states. The behavior of PC2 is different for the four investigated systems and is related to the occurrence of loop movements within the individual subunits. In 2004iNA, for example, PC2 reflects a rearrangement in the Ca^2+^ ion binding site that occurs in the monomer simulation (black) and in one subunit of the tetramer simulation (light blue).

**Figure 4.**
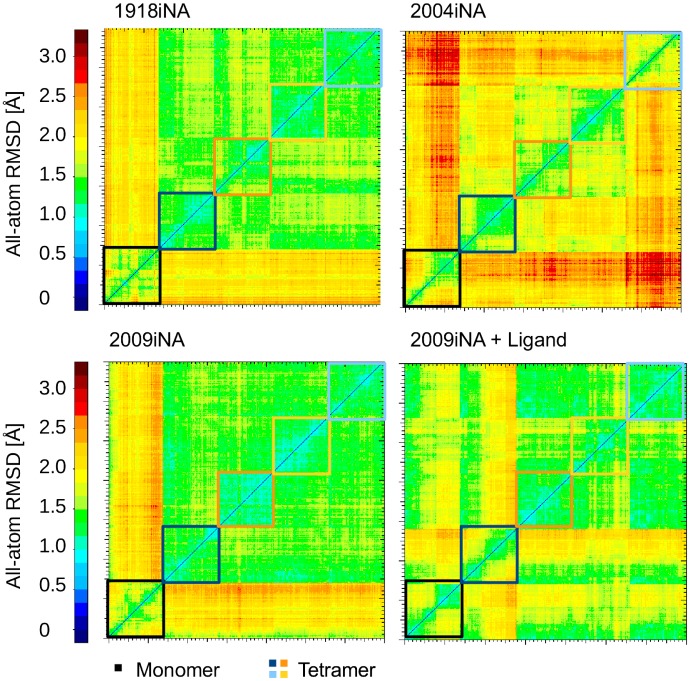
Different conformations of the binding cavities are sampled in monomer and tetramer simulations of iNAs. The heat maps of all atom RMSDs are calculated for 61 residues as observed during simulations of the monomer and the tetramer. Blue and greenish colors within the subunits of tetramer simulations indicate constrained flexibility. For the region comparing the conformations from the different subunits, green and yellow areas indicate the sampling of similar conformational space. Orange and red areas highlight substantial differences in the conformational space sampled by monomers compared to their tetrameric counterparts.

Structural comparison between the monomer and tetramer simulations by a 2D-RMSD comparison supports the findings from the PCA. In the RMSD heat map reported in Figure 4, conformations of binding site cavity residues sampled along the trajectories are compared with each other. Remarkably, the binding site geometries sampled in monomer vs. tetramer simulations differ for all four systems. Similar to observations from the 1D-RMSD analysis, the increased stability from monomer to tetramer is emphasized in Figure S3 (which compares protein backbone RMSDs). Obviously, monomers undergo more conformational transitions, whereas the individual subunits of the tetramers preserve similar conformations during simulation especially for 1918iNA and 2009iNA (Figure S3). In the presence of the ligand zanamivir, the 2009iNA monomer remains similar to the tetramer subunits for a longer part of the simulation time (about 15 ns), before exploring an alternative conformational space. One subunit of ligand-bound 2009iNA (dark blue) and one in 2004iNA (light blue) tetramer simulations samples different conformations than the other subunits, without overlap to the monomer simulations. These differences can be associated with specific loop rearrangements.

### Regional fluctuation in the iNA simulations: 110-helix and 150-loop are stabilized by assembly

Positional fluctuations calculated as B-factors for the different iNA simulations show similar patterns, as reported in Figure S4. For the catalytic domain of all simulations maxima are observed for loop regions whereas the β-sheets are more stable (Figures S4 and S5). In non-iNA Cp the non-catalytic domain with the shorter β-sheets results in a section of elevated B-factors (Figure S5).

**Figure 5.**
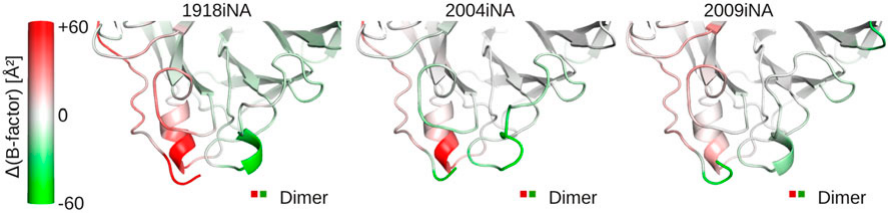
Identification of the 110-helix and the extended 150-loop as assembly sensitive regions based on three ligand-free dimer simulations. The iNA protein backbone is color coded by the differences in B-factors between the two subunits in the dimer. Protein regions colored white indicate areas of similar fluctuation in both subunits. For the 110-helix higher B-factors are observed (red coloring) in the first subunit, where this region is solvent exposed (red in dimer legend). The 150-loop is in the second subunit (green in dimer legend), resulting in negative B-factor differences (green coloring).

**Figure 6.**
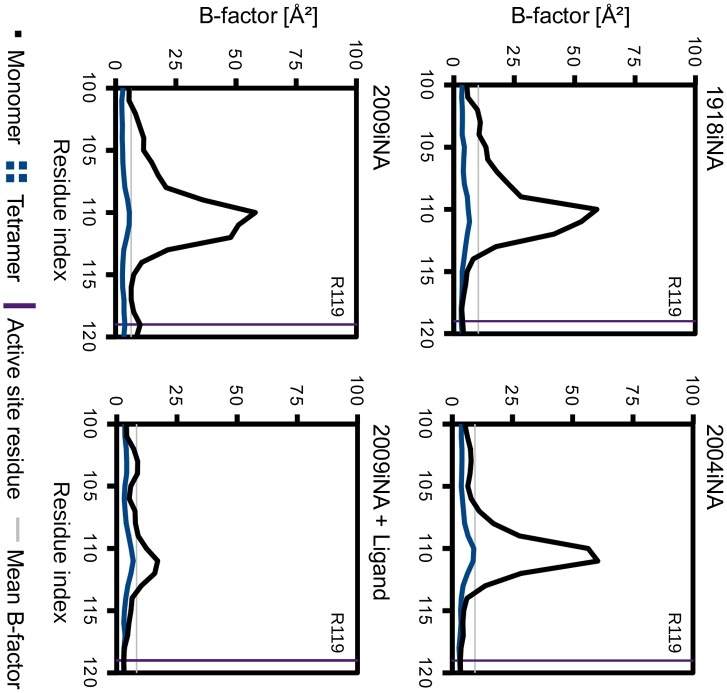
Reduction of positional fluctuations for the 110-helix in tetramer (blue averaged over four subunits) vs. monomer simulations (black; in case of 2009iNA averaged over four simulations). The 110-helix flexibility in tetramers is lower than the mean B-factor of the respective residues of all subunits in the simulations of the tetramers (light gray line). The active site residue R119 is indicated as a violet line.

Dynamics of dimer simulations provide information about the closed subunit–subunit interface as well as the respective residues in the unbound state. The comparison of distinct B-factors of the two subunits reveals two regions crucially influenced by a closed or free interface (Figure 5): The first region around Ser110, part of the 110-helix, shows increased flexibility of subunit 1 compared to subunit 2 (Figure 5, indicated in red color). In subunit 1 this helical region faces the solvent, while in subunit 2 the helix forms part of the closed interface. In simulations of the ligand-free monomer the B-factor peaks reach values of around 60 Å² on average for the ligand-free iNA simulations, which is comparable to dimer subunit 1 (Figure 6). In the monomer of ligand-bound simulation of 2009iNA, the 110-helix is stabilized in comparison to the ligand-free 2009iNA. Hence, in the latter case the difference between monomer and tetramer is not as significant as for the ligand-free simulations. However, in all four cases the B-factors for the 110-helix in the tetramer simulations remain below the average B-factor value of 10.1, 9.4, 6.5, and 8.2 Å² for the four systems. Thus, in presence of the physiological protein–protein interface, the elevated fluctuation of the 110-helix is suppressed (Figure 6).

In addition to the 110-helix, we identified a second region showing assembly state-dependent positional fluctuation. The B-factors for residues 135–155 are increased in subunit 2 when compared to subunit 1 (Figure 5, highlighted in green). Again, the solvent-exposed representative for this region shows higher fluctuations when compared to subunits with a bound interface. The presence of a neighboring subunit reduces the B-factors in the tetramer compared to the monomer simulation. In contrast to this iNA-specific insertion no assembly-dependent differences of the iNA-specific calcium binding site were identified.

### 150-loop fluctuations are sensitive to the assembly state of iNA

**Figure 7.**
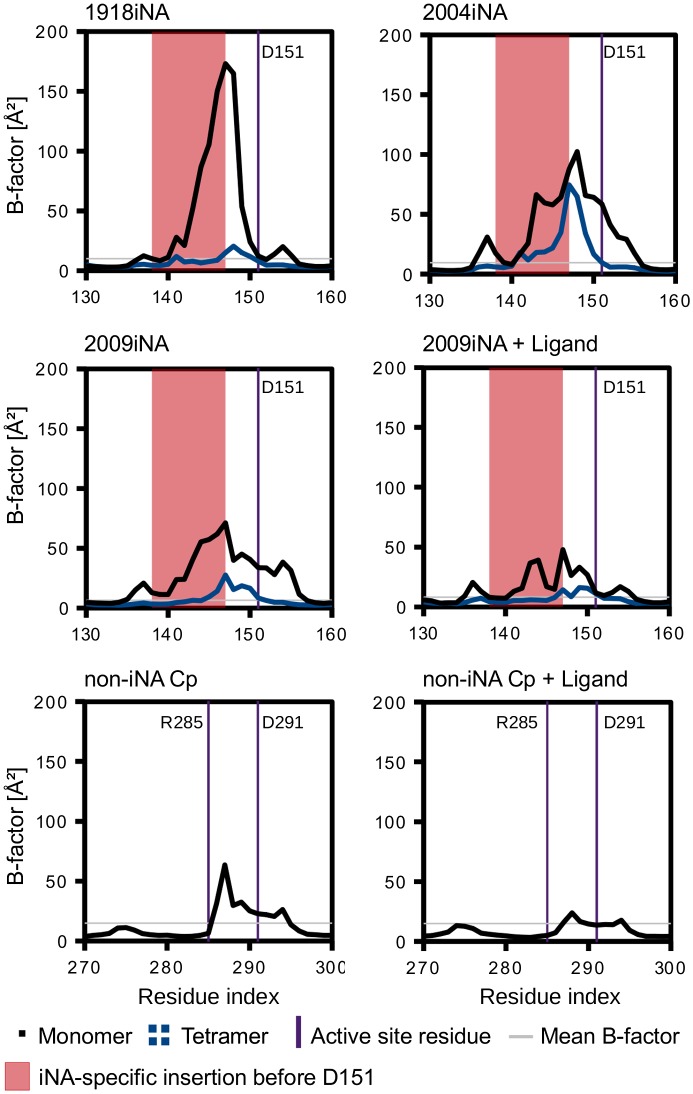
Reduction of positional fluctuations for the iNA 150-loop and equivalent region in non-iNA Cp of monomer (black) vs. in tetramer (blue averaged over four subunits). In case of 2009iNA monomer, data are averaged over four simulations.

The region of the 150-loop is of special interest as it carries the catalytic aspartic acid D151 and is known to be flexible in iNA (see Discussion). An impact of the assembly state to the flexibility has not been described yet. The comparison of the positional fluctuation between monomer and tetramer simulation indicates a clear trend (Figure 7). For iNA the monomer simulations the region of pronounced B-factor values extends over residues 135–155, while in tetramer simulations the B-factors in this region are lower. Nevertheless, a significantly higher degree of flexibility for a shorter sequence part (residues 145–151) is observed in the tetramer simulations. This is reflected in B-factor values of up to 75 Å^2^ for residue 147 in 2004iNA. In contrast to the 110-helix, no absolute conformational restriction is induced to the 150-loop by the presence of the protein–protein interface in the tetramer. The residual flexibility of the tetramer’s 150-loop exceeds these mean values for a smaller residue section than in monomer simulations. Simulations of 2009iNA were performed in presence of the inhibitor zanamivir. The ligand restricts the flexibility of the 150-loop, in particular of the active site residue D151 in comparison to the monomer simulation of ligand-free 2009iNA. The difference between monomer and tetramer simulations for the ligand-bound 2009iNA is similar to the one observed for ligand-free systems: the tetramer shows a lower flexibility and a restriction of flexibility in the part of the iNA-specific insertion.

### Ligand-binding affects 150-loop fluctuation in non-iNA in a similar way as observed for iNA tetramers

The correspondence between the catalytic residues D151 and D291 in non-iNA Cp allows a comparison of the 150-loop with the equivalent region in non-iNA Cp. This loop region in the bacterial monomer is flexible with a B-factor peak significantly greater than the mean B-factor of the whole protein. The flexibility extends over the 10 amino acids of the loop. The active site residue R285 (R119 in iNA) has an observed B-factor that is lower than this mean value. The presence of the ligand DANA in the non-iNA Cp simulation leads to a reduction of flexibility of residues in the proximity of the active site residue D291. This is without affecting the number of residues showing increased flexibility. This observation corresponds to the behavior of the tetramer simulations of 2009iNA studied with and without ligand.

### Hydrogen bond analysis of the iNA interface: Q136 mediates interface stabilization toward the active site

**Figure 8.**
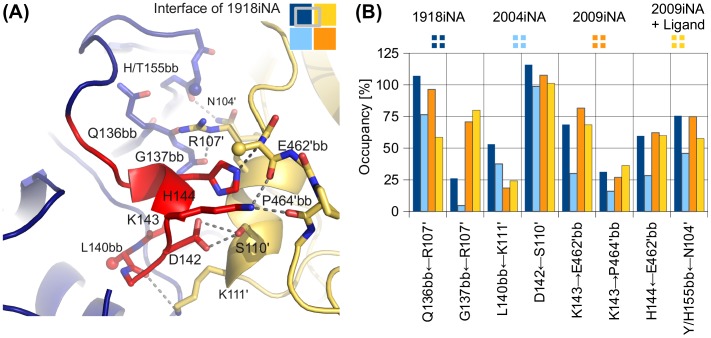
Highly populated inter-subunit hydrogen bonds in the tetramer connect the extended 150-loop with the 110-helix. (A) Hydrogen bonds (gray dashed lines) formed by residues 136–155 of 1918iNA (dark blue) to a neighboring subunit (yellow) connect to the 110-helix or the C-terminal residues E462’ and P464’. The residues of the iNA typical loop insertion are highlighted in red. Non-interacting side chains are shown as spheres at C-β. (B) Average occupancy rates show similar patterns for those hydrogen bonds in the four investigated system. Arrows are pointing from donor to acceptor residues. Residues interacting with backbone atoms are marked with bb. Occupancy rates exceeding 100% result from furcated hydrogen-bonding partners.

We analyzed the hydrogen bond occupancy rates in the tetramer simulations in order to detect important inter-subunit interactions (Figure S6). Twenty well-populated hydrogen bonds with occupancy rates >25% in at least one of the simulated systems were identified, forming an inter-subunit hydrogen bond network. Interestingly, these interactions are not equally distributed over the interface (Figures S6 and S7). A cluster of highly populated hydrogen bonds is formed by residues 136–155 (Figure 8). Interaction partners are the side chains of residues forming the 110-helix and the backbone of residues E462’ and P464’ of the neighboring subunit. The most populated hydrogen bonds are formed by residues of the iNA-specific loop insertion, between the backbone carbonyl oxygen of Q136 and Arg107’ (Q136bb←R107’), or between the side chains of D142 and S110’ (D142←S110’). The network of hydrogen bonds formed by the extended loop (covering residues 136–155) shows a remarkably similar pattern in occupancy rates for the different simulated iNAs (Figure 8(B)). In contrast, occurrences as well as occupancy rates for inter-subunit hydrogen bonds in the other interface regions show larger variations between the four investigated systems (Figure S6).

**Figure 9.**
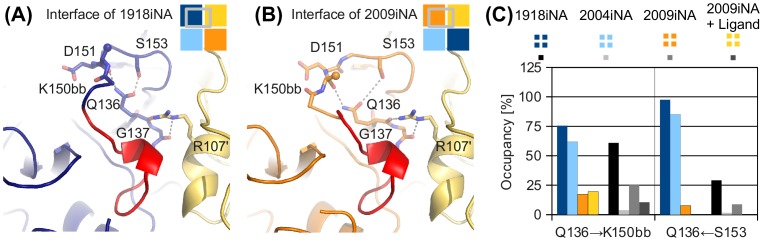
Inter-subunit interactions propagate toward the iNA active site via glutamine Q136. This conserved amino acid interacts with R107’ of the neighboring subunit (yellow) and the 150-loop residues including active site aspartic acid D151 via hydrogen bonds with the backbone oxygen of K150 and the side chain of S153 (gray lines). Different starting conformations of the 150-loop in 1918iNA (A, open 150-loop) and 2009iNA (B, closed 150-loop) are reflected in altered hydrogen bond occupancy rates for the Q136 side chain. (C) Occupancy of Q136 side chain interactions in tetramer and monomer simulations show that the tetrameric assembly state stabilizes hydrogen bonds between Q136 and two 150-loop residues, K150, and S153. Arrows are pointing from donor to acceptor residues.

We further investigated the hydrogen bond pattern of the side chain of Q136, as it extends from the interface toward the active site residue D151 (Figure 9). These interactions are also present in the monomer simulations, whereas the inter-subunit interaction of the Q136 backbone (Q136bb←R107’) is only observed for simulations with closed interfaces. In the two systems, 1918iNA and 2004iNA, the side chain amide of Q136 forms a hydrogen bond to the backbone carbonyl oxygen of K150 and the side chain hydroxyl of S153 (Q136→K150bb and Q136←Ser153; Figure 9). The oligomerization state influences the hydrogen bond occupancy rates for these systems (Figure 9(C)). There are substantial differences between the 2004iNA tetramer and monomer simulations: both hydrogen bonds are highly occupied in the tetramer, but have low occupancy rates in the monomer simulations, indicating the loss of this hydrogen bond. Compared to 2004iNA, the 1918iNA monomer shows higher occupancy rates for both hydrogen bonds in the monomer simulation. However, the hydrogen bond Q136←Ser153 shows a similar trend for high occupancy in the tetramer and a lower occupancy in the monomer. In 2009iNA, residues 147–152 show an alternative conformation (Figure 9(B)) leading to low hydrogen bond occupancy rates in the 2009iNA systems. The distance between the side chain of Q136 and the binding partners is larger than a direct hydrogen bond and corresponds to a water-mediated hydrogen bond.

## Discussion

Tetramers are the functional and catalytic active forms of iNAs; their monomer and dimer counterparts are inactive (Wu et al., 2009). This is in contrast to non-iNAs, which are active as monomers. The underlying mechanism of the iNAs’ relation between assembly state and function remains elusive so far.

Structure-guided sequence analysis of representative NAs from different biological origins, allowed the identification of two regions as typical features of iNAs (Figure 1). Earlier work on metazoal non-iNAs reported the loop between the fourth and the fifth sheet of the propeller arrangement as region of iNA-specific insertion (Giacopuzzi, Bresciani, Schauer, Monti, & Borsani, 2012). This iNA-specific insertion includes a conserved binding site for a Ca^2+^ ion (e.g. D324, G345), which is missing in non-iNAs. These residues, as well as a cysteine bridge (C318-C336) within this loop, are highly conserved among different iNA subtypes, whereas the rest of this loop insertion (residues 320–350) shows a high variability between the various iNA subtypes (Maurer-Stroh, Ma, Lee, Sirota, & Eisenhaber, 2009). For example, the residues binding a second Ca^2+^ ion in subtype N1 iNA are not conserved for N2 iNA (Figures 2(A) and S1(A)).

In this work, we highlight another loop insertion of 10 amino acids as a typical feature of iNA sequences (Figure 1). A cross-type conservation analysis with further iNAs (Maurer-Stroh et al., 2009) indicated that the insertion of residues 138–147 is an element present in all iNAs. The iNA-specific loop insertion preceding D151 directly links the active site with the tetramer interface. Remarkably, in the tetrameric context the iNA-specific loop insertion lies in spatial proximity to the neighboring subunit (Figure 2).

By comparing pairs of homologous proteins occurring in monomer and dimer states, Hashimoto and Panchenko (2010) identified oligomerization enabling regions as a mechanism of dimerization. Enabling regions are typically insertions within the sequence of the oligomerized homolog resulting in structural formations promoting the protein–protein interface between the subunits (Hashimoto & Panchenko, 2010). The identified iNA loop insertion fulfills the typical criteria of an enabling region, having a length of 10 residues with functional amino acids mediating inter-subunit contacts. The presence of an assembly enabling region in the catalytic head of N1 iNA helps to rationalize the experimental observation of functional assemblies of N1 constructs missing the transmembrane domain and the stalk domain (da Silva et al., 2013). The importance of the iNA-specific loop insertion in facilitating the iNA assembly or in stabilizing the integrity of its tetramers must be put into perspective, because free head domains of iNA tetramers tend to dissociate (Schmidt et al., 2011). Additional mechanisms of oligomerization mediated by the membrane anchoring region and the glycosylation motives in the stalk region play a role for group 1 iNA tetramer integrity and complement the mechanistic effect of the enabling loop for the tetramerization (Wu et al., 2009). In group 2 iNAs the N-glycosylation site at N200 has been postulated as an additional factor promoting tetramer stability (Air, 2012; Xu et al., 2008).

The iNA-specific loop insertion connects the protein–protein interface to the active site as it precedes the conserved catalytic D151 in the sequence. This active site residue is located in a loop region in the different NAs. The iNA-specific loop insertion results in an extended loop region of 20 amino acids, whereas in non-iNAs, the catalytic aspartic acid residues equivalent to residue D151 are embedded in shorter loop regions (around 10 amino acids).

Having highlighted the sequential and structural differences between iNAs and non-iNAs, we assume that tetramerization mainly induces differences into dynamics of the extended loop as an iNA-specific feature. In particular, the structural proximity of the iNA-specific loop insertion to the inter-subunit interface as well as to the active site with a loop region – known to be flexible in iNA subtype N1 – favors this hypothesis (Figures 2, 9(A) and (B)). Therefore, we applied MD simulations to analyze the flexibility of four iNA systems in different assembly states as well as an exemplary monomeric non-iNA.

Previously, an MD simulation study has identified the secondary structure of a sub-structural element of iNA, the 110-helix, to be critically depending on the quaternary assembly state (Amaro et al., 2007). This observation is confirmed in our study: solvent exposure of this sensitive structural element leads to an increase of B-factors (Figures 5 and 6(A)). The underlying flexibility is presumably an artificial consequence of the non-native environment. Additionally, we show altered dynamics of the 150-loop depending on the assembly state. In this region, artificial solvent exposure induces elevated flexibility which extends through residues 135–155. This observation is profound for all four investigated iNAs. In contrast, if the interface region is in native (i.e. tetrameric) state, the residues forming the iNA-specific loop insertion (135–145) are stabilized (Figures 5 and 6(B)). Experimental evidence for the dynamical sensitivity of these two regions toward protein assembly can be found in the recently resolved structures of the NA-like N10 protein (Li et al., 2012). The regions equivalent to the 110-helix (residues 103–112) and 150-loop (residues 140–155) are only resolved in the crystal showing an iNA-typical tetramer assembly. In the monomer form of N10, the regions are unresolved due to high flexibility (Li et al., 2012).

An influence of assembly state on the 150-loop dynamics is of special interest: two conformations of residues 147–152 have been observed in crystal structures of group 1 iNA; an open state found in most N1 apo structures (Figure 9(A)) and a closed state found in ligand-bound X-ray structures and for the apo structure of 2009iNA (Figure 9(B)) (Li et al., 2010; Russell et al., 2006). MD simulations indicated a high- intrinsic mobility of this loop region, including the active site residue D151 (Amaro et al., 2007, 2009, 2011; Grienke et al., 2010). In ligand-free structures the conformation of the 150-loop gives access to an additional cavity next to the binding site, which is therefore referred to as “open state”. Larger iNA inhibitors are assumed to bind to that area (Cheng et al., 2008; Grienke et al., 2010; Kirchmair et al., 2011; Rudrawar et al., 2010; Russell et al., 2006). Additionally, the open state conformation of the 150-loop was recently associated with group 1 specificity of the iNA inhibitors zanamivir and laninamivir (Vavricka et al., 2011). Both inhibitors have bulky guanidinium groups buried beneath the 150-loop. In subtype N2, a group 2 NA, alternative conformations of the 150-loop were structurally characterized very recently (Wu et al., 2013). Also, in human NA (non-iNA Hs) the loop embedding the equivalent catalytic aspartic acid (D46) is flexible, as it is unstructured in the apo state and only resolved at X-ray level upon ligand binding (Chavas et al., 2005). This flexibility is assumed to play a role in ligand recognition (Chavas et al., 2005). In the non-iNA of *C. perfringens* the corresponding D291 side chain crystallized with two alternative conformations (Newstead et al., 2008). An MD study on the hemagglutinin-NA of the parainfluenza virus identified a corresponding loop which closes upon ligand-binding (Winger & von Itzstein, 2012). Altogether, there is increasing evidence that the flexibility of the 150-loop or the corresponding region in other NA enzymes is a common feature of NA with a functional role for the NA activity. We observe an increased flexibility of the iNA-specific insertion (138–147) and adjacent residues in the non-native monomer state (Figure 7). In consequence, the dynamics of the 150-loop with the catalytic D151 are modulated by the NA assembly state. The restriction in the tetramer is not as rigorous as for the 110-helix as the residues 147–151 surmount the average fluctuation calculated for the whole protein. The corresponding region in the non-iNA Cp shows a pronounced flexibility, especially when simulated in the native monomer state without ligand. However, flexibility is limited to five residues before and three residues after the catalytic D291. This situation is more similar to the tetramer state of iNA and different to the monomer state of iNA, where the flexible region extends over around 20 residues. We assume that the conformational restriction by tetramerization has a balancing influence on the flexibility of the 150-loop. The presence of a ligand restricts the 150-loop flexibility in the 2009iNA and in the non-iNA Cp. Previously, ligand binding was discussed as a factor influencing the 150-loop dynamics and investigated in monomer MD simulations observing similar restriction effects for NA inhibitors such as zanamivir (Greenway, LeGresley, & Pinto, 2013). However, the effects we observe for ligand binding are not as striking as the effect for tetramerization. The strong effect of the bioactive assembly on the 150-loop dynamics suggests that results from tetramer simulations are more relevant in regard to the interpretations of factors influencing the 150-loop dynamics (e.g. Amaro et al., 2011).

We investigated the hydrogen-bonding patterns formed between the subunits of the tetramer simulations to study the mechanism behind the sensitivity of iNAs to their assembly state. Thereby, we identified a set of prominently occupied hydrogen bonds linking residues 135–155 (including the iNA-specific loop insertion and the 150-loop) with 110-helix and the backbone atoms of the C-terminal loop (Figure 7). These inter-subunit contacts contribute to the stability of the protein–protein interface and corroborate the hypothesis that the iNA-specific loop insertion functions as enabling insertion in the tetramer assembly. Additionally, this stabilization effect leads to substantially different motion patterns and, in consequence, to distinct conformations of both interface region and binding site. Interestingly, in the recently described open state conformation of N2 iNA, an inter-subunit contact was shown to be critical for the stabilization of this protein conformation (Wu et al., 2013). In N2, D142 forms a hydrogen bond to R107’ in the adjacent subunit (D142←R107’), thereby stabilizing the open conformation of the 150-loop via a water-mediated hydrogen bond with H150. For the N1 systems investigated in this study, we propose that a propagation to the active site is mediated, e.g. by Q136, which forms an inter-subunit hydrogen bond with R107’ and interacts with active site residues (Figure 9). For the simulations of 1918iNA and 2004iNA (both with an open 150-loop conformation), we show that the oligomerization state influences the occupancy of the hydrogen bond between Q136 and the backbone peptide connecting K150 with the catalytic D151. Thus, we surmise that Q136 plays a role as connecting element in the structural communication between interface and active site.

Besides this potential mechanism of propagation, we demonstrated the overall impact of the assembly state on the conformational sampling, by PCA and the comparison of 2D-RMSDs. In the PCA study of Amaro et al. (2007), only the subunits from the tetramer simulation were considered. As in this study, our results show that individual subunits of the tetramer simulations have some distinct individual conformational behavior. In 2004iNA and 2009iNA, PC2 separates the fourth subunit and the monomer due to the high flexibility in the loop 340–345 and 245–250, respectively. However, the eigenvector with the highest contribution to the variation in the covariance matrices of sampled conformations reflects the difference between the assembly states in all iNA systems.

The propagation of the stabilization effect is reflected in different regions of conformational space accessible for monomer and tetramer (Figures 3 and 4). As a consequence of local changes in protein fluctuation, the stabilization induced by the assembly state is extended to the whole protein (Figure S3) and has a substantial effect on the geometry of the active site (Figure 4).

Consequently, we propose that stabilization of the overall protein fold as well as maintaining the active site geometry by the tetramerization is the main cause for iNAs to be only active as tetramers. Thereby, the role of residues 138–147 as mediator of the assembly dependency is outstanding, since we identified this extension in the 150-loop as an iNA-specific feature that is not present in the monomeric analogs occurring in other organisms.

A similar impact of tetramerization has been observed for lactate dehydrogenase (LDH), where comparative MD simulations showed that description of the active site geometry depends on the setup of the environment (Schmidt & Gready, 1999) This study pointed out that stabilization of the enzyme active site via an inter-subunit contact of the α1G-α2G-helix adheres enzymatic inactivity of LDH monomers. Analogous to this hypothesis, we postulate that tetramerization and in particular the contacts formed between the 110-helix and the iNA-specific loop insertion stabilize the conformation of the active site. This explains the experimentally observed assembly dependency of the iNA activity by changes in dynamics and structural rearrangements upon dissociation of the tetramer.

Nevertheless, some questions about the causative reason for tetramer assembly of iNAs remain open. While LDH tetramerization has been observed to allow the formation of heteromeric enzyme isoforms with altered catalytic properties to fulfill complementary roles in different tissues, for iNAs no obvious beneficial effect of specific assembly states has been identified so far. Taking into account the importance of iNAs as antigenic factors, we speculate that the driving force for tetramerization may be reduction of the solvent-exposed surface, which function as antibody binding sites (Colman, Varghese, & Laver, 1983; Varghese et al., 1983). Homo-assembly reduces the solvent-exposed regions and protects residues within the interface from interaction with the host’s antibodies.

## Conclusions

The active forms of viral iNAs are homotetramers, in contrast to NAs from other biological sources, which are active as monomers. Based on MD simulations of N1 from avian 2004, pandemic 1918, and pandemic 2009 influenza A strains, we show that the different NA assembly states have characteristic 150-loop dynamics. These fluctuations are heavily influenced by the presence or absence of the hydrogen-bonding network between two iNA subunits formed by the residues of the 110-helix and the 150-loop. The importance of the link between 150-loop and homotetrameric assembly is also established by the fact that the analogous region in monomeric non-iNAs is shorter and lacks the iNA-specific loop insertion of around 10 amino acid residues.

**Table:** 

**Abbreviations**	
NA	Neuraminidase
iNA	Influenza neuraminidase
Non-iNA	Non-influenza neuraminidase
MD	Molecular dynamics
PDB	Protein data bank
Mv	*Micromonospora vidifaciens*
Cp	*C. perfringens*
Sp	*Streptococcus pneumoniae*
Hs	*Homo sapiens*
Af	*Aspergillus fumatus*
Tc	*Trypanosoma cruzi*
DANA	2-deoxy-2,3-dehydro-N-acetyl neuraminic acid
RMSD	Root mean square deviation
PCA	Principal component analysis

## Supplementary material

Supplementary material dealing with (i) pair-wise sequence identities of NAs (Table S1), (ii) setup of simulated systems (Table S2), (iii) structures of NAs of other biological systems (Figure S1), (iv) backbone RMSD fluctuation (Figure S2), (v) backbone RMSD means and standard deviations for simulated systems (Table S3), (vi) two-dimensional RMSD comparison between monomer and tetramer simulations (Figure S3), (vii) backbone B-factors for all simulated iNA systems (Figure S4), (viii) backbone B-factors for simulated monomer of non-iNA Cp systems (Figure S5), (ix) inter-subunit hydrogen bond occupancy values in iNA tetramer simulations (Figure S6), and (x) two clusters formed by the inter-subunit hydrogen bond in the interface (Figure S7) is available online at http://dx.doi.10.1080/07391102.2013.855142.

## Funding

The research of the manuscript was supported by funding of the Austrian Science Fund FWF: projects “Targeting Influenza Neuraminidase” (P23051) and “Natural Lead Structures Targeting Influenza” (P24587). This work was supported by the Austrian Ministry of Science BMWF as part of the research platform Scientific Computing at University of Innsbruck. MS is financially supported by the European Social Fund (ESF) and the Thuringia Ministry of Economy, Work and Technology (TMWAT) with the project 2011FGR0137. SvG thank the University of Innsbruck for a young scientist grant.
